# Tunneling Conductivity and Piezoresistivity of Composites Containing Randomly Dispersed Conductive Nano-Platelets

**DOI:** 10.3390/ma7042501

**Published:** 2014-03-28

**Authors:** Amirhossein Biabangard Oskouyi, Uttandaraman Sundararaj, Pierre Mertiny

**Affiliations:** 1 Advanced Composite Materials Engineering Group, Department of Mechanical Engineering, University of Alberta, Edmonton T6G 2G8, AB, Canada; E-Mail: biabanga@ualberta.ca; 2 Department of Chemical and Petroleum Engineering, University of Calgary, Calgary T2N 1N4, AB, Canada; E-Mail: ut@ucalgary.ca

**Keywords:** nanocomposites, electrical properties, modeling, piezoresistivity effect

## Abstract

In this study, a three-dimensional continuum percolation model was developed based on a Monte Carlo simulation approach to investigate the percolation behavior of an electrically insulating matrix reinforced with conductive nano-platelet fillers. The conductivity behavior of composites rendered conductive by randomly dispersed conductive platelets was modeled by developing a three-dimensional finite element resistor network. Parameters related to the percolation threshold and a power-low describing the conductivity behavior were determined. The piezoresistivity behavior of conductive composites was studied employing a reoriented resistor network emulating a conductive composite subjected to mechanical strain. The effects of the governing parameters, *i.e*., electron tunneling distance, conductive particle aspect ratio and size effects on conductivity behavior were examined.

## 1. Introduction

The development of nano-scale electrically conductive fillers and associated low cost fabrication methods has stimulated considerable interest in employing such particles to render otherwise insulating polymers conductive. Conductive nano-fillers, such as graphene platelets and exfoliated graphite, were found to impart significant conductivity and piezoresistivity behavior to polymers, which makes such material systems an excellent choice for electrical applications, such as strain sensors. Nano-platelet based conductive polymers have been the subject of a number of experimental studies [1–9]. Generally, these works were devoted to developing efficient fabrication methods and evaluate the conductivity and critical filler volume fraction at which a composite exhibits a transition from insulating to conductive behavior. Percolation theory has been a successful tool for describing the transition behavior of insulating polymers filled with conductive inclusions [10–14]. According to percolation theory a critical filler volume fraction exists at which a percolation network is formed that renders a composite conductive. The critical filler volume fraction at the onset of a percolation network is called the percolation threshold. Monte Carlo simulation techniques have widely been employed to evaluate the percolation threshold and conductivity behavior of conductive composites [10–16]. A large number of numerical and analytical investigations have been devoted to insulating polymers filled with spherical and stick-shaped conductive inclusions. The majority of works related to conductive composites with platelet fillers has been experimental, and only few numerical and analytical studies on this subject are described in the technical literature [17–24]. Some researchers used Monte Carlo simulation techniques to evaluate the conductivity of platelet based conductive polymers. Hicks *et al.* [17] developed a Monte Carlo-based computational model to predict the tunneling percolation behavior of a resistor network formed by rectangular conductive sheets that are oriented parallel to a substrate. Li and Morris [18] developed a two-dimensional (2D) computational percolation model to investigate the critical filler content and conductivity behavior for an adhesive filled with conductive flakes. They assumed the inclusions to be rectangular plates that are dispersed in a 2D space. The excluded volume method is another techniques employed for studying the percolation threshold of conductive platelet based composites. Xia and Thorpe [19], for example, investigated the percolation behavior of overlapping ellipses randomly located in a plane using the excluded volume method. Modeling based on the excluded volume method however has its limitations as it is unable to predict the effect of tunneling distance on the critical volume fraction. Consequently, for cases in which tunneling distance plays an important role in the conductivity behavior of composites (e.g., when particle dimensions and electron hopping distance become comparable in scale), excluded volume method based modeling is no longer efficient and cannot be employed to study the tunneling conductivity in conductive composites.

Yi and Tawerghi [20] carried out a numerical Monte Carlo based study to evaluate the percolation threshold for 2D interpenetrating plates, *i.e*., circular, elliptical, square, and triangular plates dispersed in three-dimensional (3D) space. It was shown that noncircular geometries tend to produce higher percolation thresholds. The percolation behavior of penetrable disks has been the subject of several other studies [21–23]. Vovchenko and Vovchenko [24], for example, predicted the percolation threshold of composites filled with intersecting circular disks. Otten and Schoot [25] developed an analytical approach to investigate the percolation behavior of polydisperse nanofillers, specifically focusing on conductivity of needle-like filler nanocomposites. They also briefly investigated the percolation behavior of platelet-based composites. However, their modeling approach was subject to certain limitations, that is, the platelet thickness and the so-called tunneling decay length have to be of the same order of magnitude, and the diameter of disk-like fillers needs to be much larger than the disk thickness (tunneling decay length corresponds to inverse of the square root of the insulator barrier height in the present study). For monodisperse cases their model predicts the percolation threshold to be proportional to the ratio of filler thickness to decay length; interestingly, the percolation threshold was independent of the filler diameter. Ambrosetti *et al.* [26] conducted a numerical study to investigate the percolative properties of a system of hard oblate ellipsoids of revolution surrounded with soft penetrable shells. For higher aspect ratios, oblates approach a disk-like geometry but still deviate from an ideal circular disk. As described in a latter part of the present text (Section 3.3), data from [26] was employed for comparison to platelet filler based tunneling percolation problems. In a later work by Ambrosetti *et al.* [27], their initial study was expanded to evaluate the conductivity of percolation networks formed by ellipsoids of revolution dispersed in an insulating matrix. Mathew *et al.* [28] conducted a Monte Carlo study on the percolation of hard platelets in a 3D continuum system. They represented the hard platelets by cut-spheres, which vary in shape from a platelet geometry. In their model, platelet-like geometries were generated through intersecting a sphere with two parallel planes at equal distance from the equatorial plane. Their findings indicate that lower aspect ratios are associated with a lower percolation threshold. Note that the majority of related works neglected the condition of impenetrable disks, and only a few studies in the technical literature are devoted to the percolation behavior of impenetrable hard disk-like inclusions. To the best of the authors’ knowledge, research on this subject matter is limited to the last four studies mentioned above, which focus mainly on the percolation properties. Conductivity behavior in terms of a critical exponent describing post-percolation behavior and, more importantly, piezoresistivity behavior have been out of their scope. Moreover, in these studies, circular disks were approximated by ellipsoids or cut-spheres. Hence, studies on the tunneling percolation behavior of impenetrable circular disks in 3D space are lacking.

The limited quantity of published works related to the modeling of platelet based conductive polymers, and limitations associated with the employed techniques motivated the present authors to conceive an alternative approach for modeling the electrical behavior of polymers filled with conductive nano-platelet fillers. Published research on the electrical properties of nano-platelet based nanocomposites has further been limited to studying the electrical conductivity of polymers with exfoliated sheets with in-plane dimensions varying from a few to several hundred micrometer and thicknesses ranging from a few to several nanometers. To achieve further enhancements in composite properties the synthesis of submicron size single layer graphene sheets may be an attractive proposition, and information on the percolation and electrical behavior of such reinforced polymers is therefore desirable. The latter aspects are also addressed in the present contribution.

A 3D Monte Carlo model was developed to study the percolation, conductivity and piezoresistive behavior of composites filled with randomly dispersed impenetrable conductive nano-disks. In the present study a Monte Carlo model was first developed to form a representative volume element filled with randomly dispersed nano-platelet conductive inclusions. In a second stage a 3D finite element based resistors network model was used to analyze the conductivity behavior of nano-platelet based conductive polymers. Previous studies have shown that conductivity of such polymers for volume fractions greater than the percolation threshold can be described by a power-low expression [29], *i.e.*:

$$\rho \propto {\left(V-V_{\text{p}}\right)}^{s}$$ (1)

where *V*_p_ is the percolation threshold, *V* is the volume fraction of the dispersed inclusions, and ρ and *s* are the resistivity and a critical exponent respectively. Parameters in Equation (1) were determined through curve fitting using the results obtained from finite element modeling. The resistor network model was also used to study the piezoresistive behavior for reoriented and updated conductive platelets when a conductive composite is subjected to mechanical strain.

## 2. Formulation

### 2.1. Random Nano-Platelet Generation

The developed 3D continuum Monte Carlo model is based on conductive inclusions that are randomly dispersed inside a cubic representative volume element (RVE) with side length *L*. Note that further details on the model described herein can be found in [30]. Conductive platelets were assumed as impenetrable circular disks with diameter *D* and thickness *t*. Circular disks were randomly generated and uniformly dispersed inside the RVE. Three random numbers (*x*_c_,*y*_c_,*z*_c_) were generated between (0,*L*) to establish the coordinates of the center of each disk. The random number generator algorithm developed by Matsumoto and Nishimura [31] was used for random number generation. Cornejo Díaz *et al.* [32] confirmed the suitability of this algorithm for Monte Carlo applications. In order to determine the disk orientation, *i.e*., the normal to the plane containing a disk, a point from the surface of a unit sphere was chosen employing the method presented by Marsaglia [33]. The point thus selected represents the end point of the normal vector with origin located at the center of the disk. Since, in this study, nano-platelets were assumed to be impenetrable rigid disks, the geometric feasibility of any generated disk needed to be validated so that only those disks were added to the RVE that have no intersection with existing disks. The intersection of two intersecting disks is a segment of a line, which is determined by the cross product of the normal vectors of the disk planes (***L*** = ***n*_1_** × ***n*_2_**). Two disks (*i* and *j*) are intersecting when the following inequality is satisfied:

$$\sqrt{R^{2}-{\bar{o_{i}p_{i}}}^{2}}+\sqrt{R^{2}-{\bar{o_{j}p_{j}}}^{2}}\leq \bar{p_{i}p_{j}}$$ (2)

where *o_i_* is the center of the *i*-th disk and *p_i_* is a point on line ***L*** that is corresponding to the shortest distance of *o_i_* from line ***L***.

### 2.2. Electrical Conductivity, Clusters and Percolation Network

Electrical connection between two conductive inclusions arises from two different mechanisms, *i.e*., (i) mechanical contact between conductive particles; or (ii) electron tunneling effects. Based on quantum mechanical tunneling theory, electrical current can flow under certain conditions through an insulator material. In other words, it can be assumed that a pair of conductive inclusions dispersed in an insulator matrix is electrically connected by a resistor formed by the matrix so that electrons can passage from one inclusion to the adjacent one. It was shown in different studies that tunneling conductivity plays the dominant role in the conductivity of insulating polymers filled with conductive inclusions [34]. For strongly conductive fillers the intrinsic filler conductivity has no appreciable contribution toward the nonlinear current-voltage behavior of nanocomposites, with nonlinearity arising from tunneling effects. Experimental research works devoted to studying the current-voltage behavior of carbon nanotubes (CNT) based [35,36] and graphene nano-platelet based [37,38] conductive nanocomposites indicate that nonlinear current-voltage behavior is more pronounced for disk-shaped fillers. The significant dependency of conductivity on voltage in circular disks indicates that conductivity is indeed governed by tunneling mechanisms. As shown by Simmons [39], electron tunneling resistivity can be described by Equation (3) for a low-voltage range when *e*Δ*V* << λ, where Δ*V* is the electrical potential, *e* is the quantum of electricity and λ is the barrier height of the insulator.

$$\rho_{\text{tunl}}=\frac{h^{2}}{e^{2}\sqrt{2m\lambda }}\text{exp}\left(\frac{4\pi d}{h}\sqrt{2m\lambda }\right)$$ (3)

where *h* is Planck constant, *m* is the mass of an electron and *d* is the tunneling distance.

**Figure 1.**
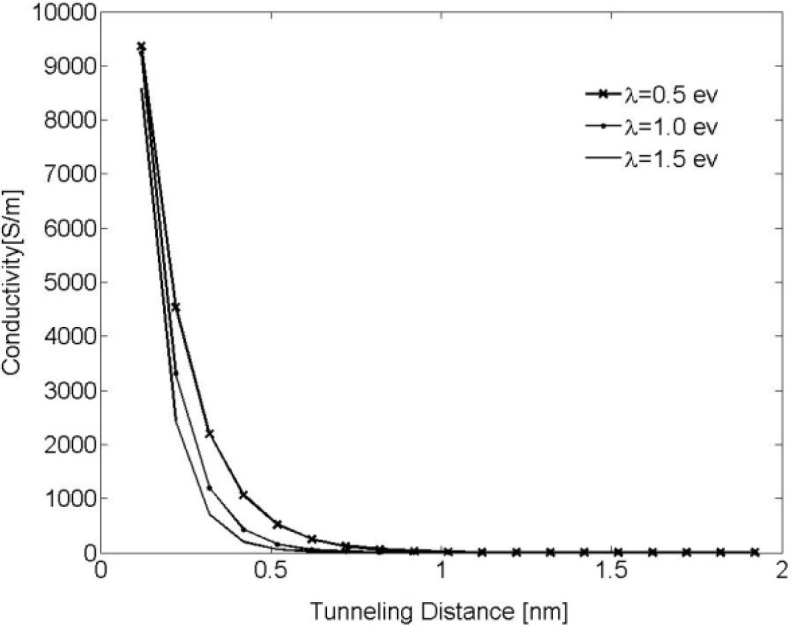
Tunneling conductivity *vs*. tunneling distance for electrons passing through an insulator matrix for different insulator barrier heights λ.

Equation (3) was numerically evaluated for different values of λ (λ = 0.5, 1.0 and 1.5 eV) and results are illustrated in Figure 1. It can be observed that tunneling conductivity (*i.e*., the inverse of resistivity) drastically decreases for an increasing tunneling distance. It can, thus, be concluded that a cut-off distance can be approximated at which resistors with length greater than this distance have no appreciable contribution to the overall conductivity of the nanocomposite. This cut-off distance is called electron-tunneling distance. In the developed model, nano-platelets with neighboring distance less than the tunneling distance are labeled a cluster. A cluster connecting two parallel faces of the RVE is called a percolation network, which enables electrical current to flow through the RVE, thus, rendering the nano-composite conductive.

**Figure 2.**
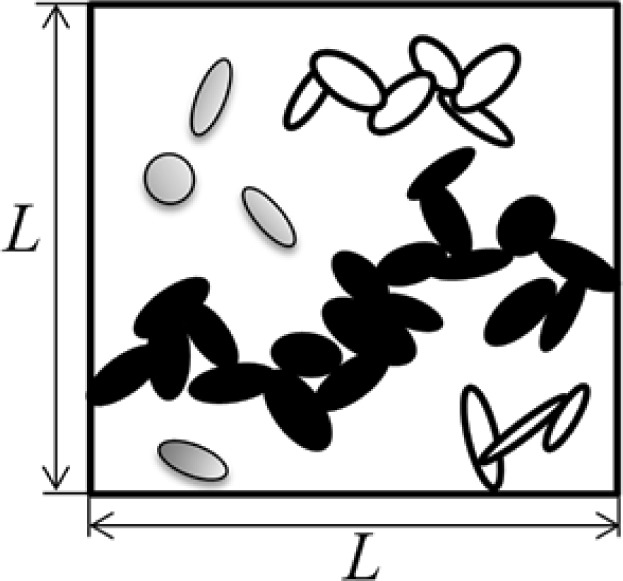
Schematic of representative volume element with individual nano-platelets (gray), and platelets forming clusters (white) and a percolation network (black).

**Figure 3.**
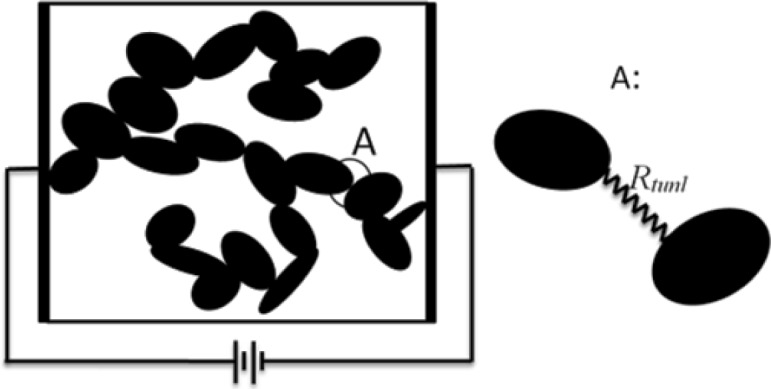
Schematic of three-dimensional percolation network modeled by tunneling resistors (A).

The volume fraction of the conductive particles at the onset of the percolation network is called percolation threshold. This concept is illustrated by the schematic shown in Figure 2. It was assumed that current flow between two conductive inclusions would occur along the shortest possible path in between them. It is therefore necessary to determine the shortest distance between the inclusions. The algorithm developed by Almohamad and Selim [40] was employed in the present modeling approach to compute the shortest distance between two circular disks in 3D space. In order to estimate the conductivity of a conductive composite a 3D network of resistors was formed (see schematic in Figure 3). As such, randomly dispersed conductive nano-platelets were modeled as circular disks generated and randomly dispersed inside the RVE using the methods described above, and disks in clusters were mutually connected by tunneling resistors which resistivity was approximated using Equation (3). Disks were assumed to be in contact with each other when the shortest distance between their center planes is less than the thickness of the disks. Finite element modeling was employed to evaluate the conductivity of a resistor network representing the nanocomposite.

### 2.3. Piezoresistivity Effects

**Figure 4.**
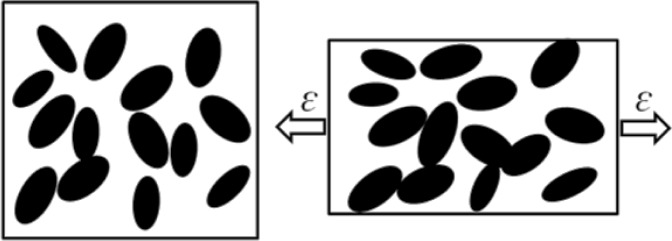
Schematic of nano-platelet composite subjected to tensile strain.

Subjecting a polymer with conductive inclusions to mechanical strain, as shown in Figure 4, is often accompanied by a change in resistivity. This behavior is known as the piezoresistivity effect, which is an interesting characteristic of conductive nano-composites rendering them good candidates for sensor applications. Generally, there are two main reasons for piezoresistive behavior of nano-composites, *i.e*., (i) changes in interparticular distance; and (ii) reorientation of conductive particles. Piezoresistivity of nanocomposites with conductive inclusions has been the subject of several numerical and analytical works [17,41–43]. In the present study, the described framework of Monte Carlo simulation and finite element modeling were employed to evaluate the piezoresistive behavior of conductive composites with nano-platelets. Most conductive nano-fillers have superior mechanical properties in comparison to the matrix. As a consequence their deformation was neglected for a nanocomposite being subjected to mechanical strain, and conductive nano-platelets were assumed to undergo rigid body motion only. Note that nano-platelets such as graphene may be wrinkled or crumpled when dispersed in a host polymer. The probability of being non-planar increases as the size of the nano-disk is increasing. Since this study was conducted for small-size nano-disks, it was assumed that disks remain planar during dispersion and mechanical loading. Further work may be conducted to evaluate the effect of wrinkling and bending on the nanocomposite electrical behavior. The new position and orientation of the nano-platelets in the deformed matrix (for uniaxial tensile strain) were determined using affine transformation utilizing the formulation developed by Pham [43]. Filler reorientation and displacement inside the polymer matrix is also governed by lateral mechanical strain, which is induced by Poisson’s ratio effects. For numerical simulations the Poisson’s ratio was set at 0.33, which was found to be the mean value reported in the literature for the Poisson’s ratio of nano-platelet based conductive composites. A 3D resistor network was then re-developed for the updated nano-platelet structure, and aforementioned finite element modeling was employed to evaluate the conductivity of the strained nano-composite.

## 3. Results and Discussion

The computational code for the Monte Carlo simulation was developed in the FORTRAN language to numerically investigate the 3D continuum percolation problem of conductive nano-disks. Considering the high computational cost of the Monte Carlo simulation, multiple processor computation was employed to allow for rapid code execution on a Linux cluster.

The developed model was evaluated numerically considering a polymer matrix such as epoxy containing conductive nano-disk filler comprised of graphene platelets. Hence a constant nano-disk thickness of 0.34 nm was assumed, which is equal to the thickness of a single graphene layer [44]. Considering the high conductivity of graphene predicted by experimental and numerical studies [45], it can reasonably be said that tunneling resistance dominates the conductivity properties of graphene nanocomposites. Hence, the electrical potential difference across graphene sheets was herein assumed negligible compared to the potential drop in the tunneling resistors. Ignoring the resistivity of the nano-disks in the present model also resulted in a considerable decrease in computational cost. The RVE size was set to be eight times *2R + d_t_*, which was found large enough to minimize any errors introduced by finite size effects (*d_t_* and *R* are the tunneling cutoff distance and nano-disk radius, respectively).

### 3.1. Effective Electron Tunneling Distance

**Figure 5.**
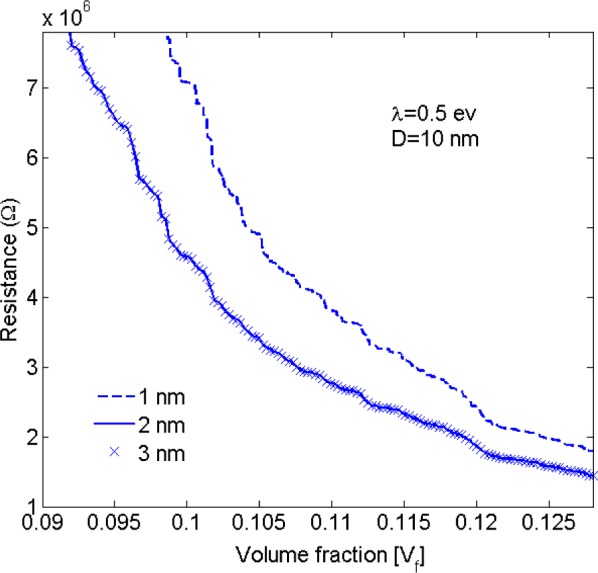
Effect of tunneling cut-off distance (1 nm, 2 nm and 3 nm) on the resistivity of nano-platelet composites for an insulator barrier height of λ = 0.5 eV and a platelet diameter of *D* = 10 nm.

As discussed earlier, a cut-off distance can be assumed at which the effect of tunneling resistors with length greater than this distance is negligible. The conductivity of nanocomposites was evaluated for different values of electron tunneling distance. Considering the results shown in Figure 5 it was concluded that for a barrier height of 0.5 eV (for epoxy polymer [42]), tunneling resistors with length greater than 2 nm have no appreciable contribution to the conductivity of the nanocomposite. It the present study the tunneling distance was, thus, assumed to be 2.5 nm. This value is conservatively larger than values given in the literature (e.g., 1.4 nm in [46]), ensuring that essentially all tunneling paths that may contribute toward nanocomposite conductivity have been taken into account.

### 3.2. Effect of Matrix Electrical Properties on Nano-Composite Conductivity

**Figure 6.**
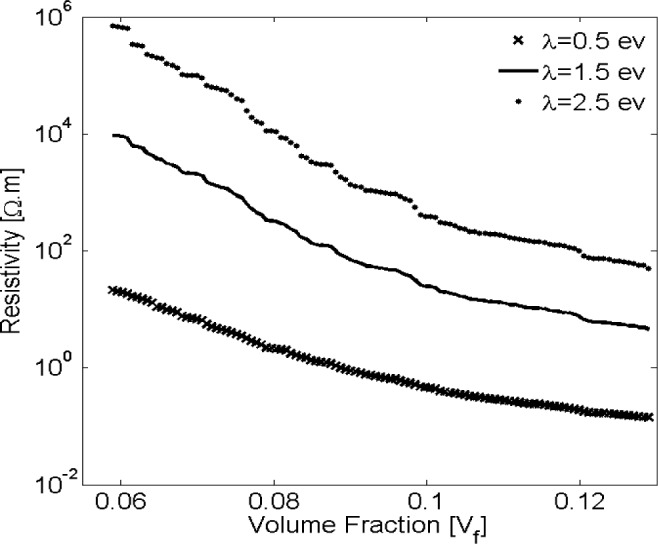
Effect of insulator barrier height λ on the resistivity of conductive nano-platelet composites plotted *vs.* filler volume fraction for a platelet diameter of *D* = 10 nm.

It was stated previously that the polymer matrix chiefly affects the electrical resistivity of a nano-composite, especially for lower filler volume fractions. As such, the contribution of matrix electrical properties, *i.e*., the barrier height λ should be investigated. Electrical resistivity was evaluated for three different barrier heights, *i.e*., 0.5, 1.5 and 2.5 eV, representing typical values reported as height of barrier for epoxy [42] filled with randomly dispersed nano-platelets. Results illustrated by Figure 6 indicate that the matrix barrier height strongly affects the nano-composite conductivity. For example, for a filler volume fraction of 7.5% the electrical resistivity of a nano-composite with λ = 1.5 eV is more than two orders of magnitude greater than for a matrix with λ = 0.5 eV.

### 3.3. Percolation Threshold and Electrical Conductivity

**Figure 7.**
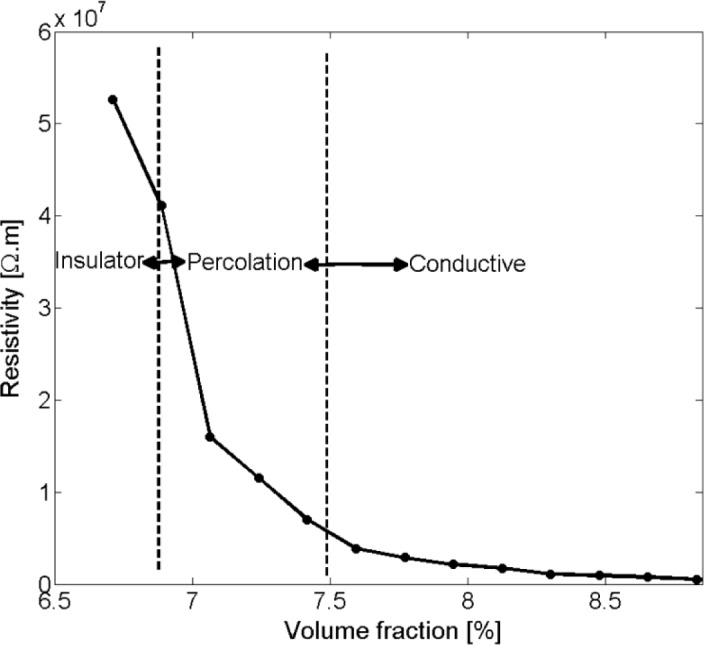
Graph defining the percolation region depicted as nano-platelet composite resistivity *vs.* filler volume fraction for a platelet diameter of *D* = 100 nm.

Figure 7 illustrates the resistivity of nanocomposites with respect to changes in nano-disks volume fraction. A critical volume fraction is recognizable at which a sharp drop in nanocomposite resistivity occurs. This region was considered the formation of a percolation network.

**Figure 8.**
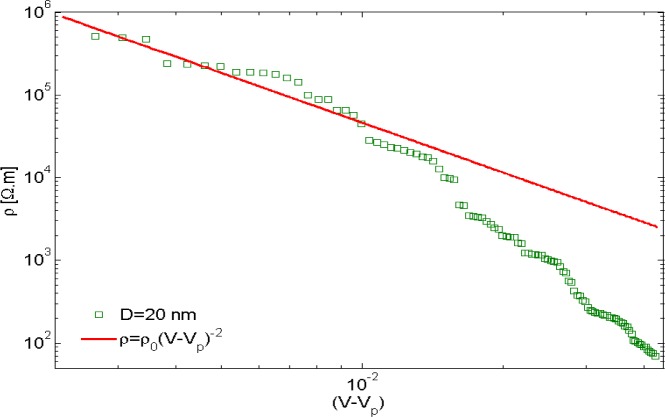
Deviation of nano-platelet composite resistivity from a universal power-law description with *s* = 2 for increasing filler content.

**Figure 9.**
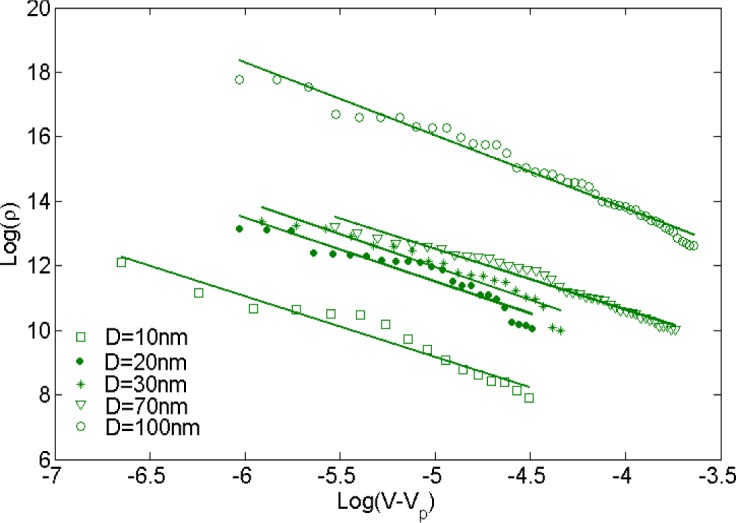
Nano-platelet composite resistivity *vs.* filler volume fraction and best-fit curves for post-percolation power-law description for filler concentration near the percolation threshold (*V*_p_, 1.4*V*_p_).

**Figure 10.**
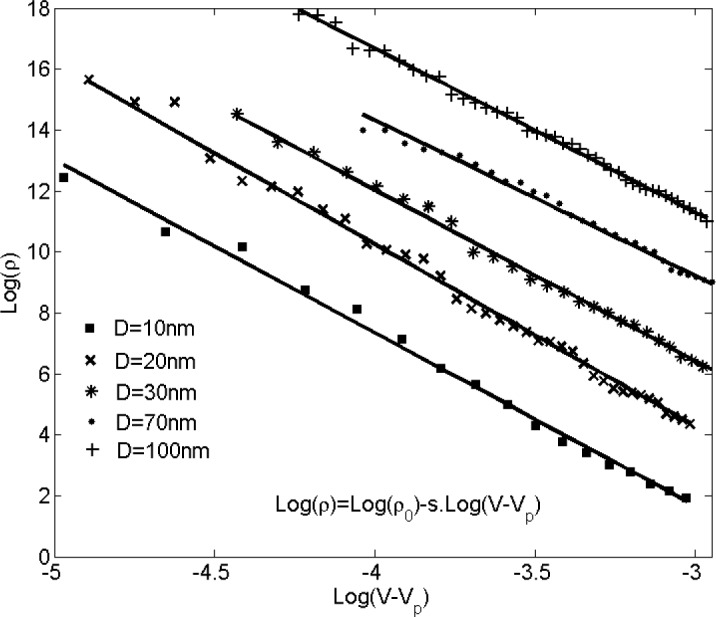
Nanocomposite resistivity *vs.* filler volume fraction and best-fit curves for a post-percolation power-law description (percolation region and erratic points are considered data outlier).

**Figure 11.**
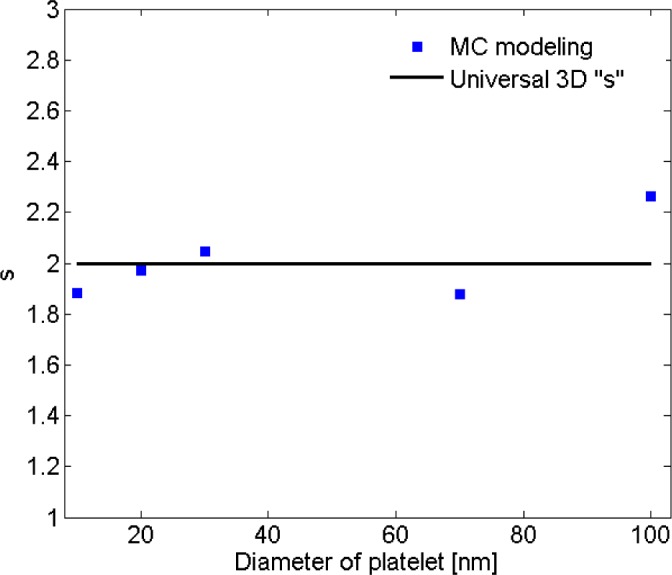
Values for the critical exponent, *s*, for data in the percolation region.

**Figure 12.**
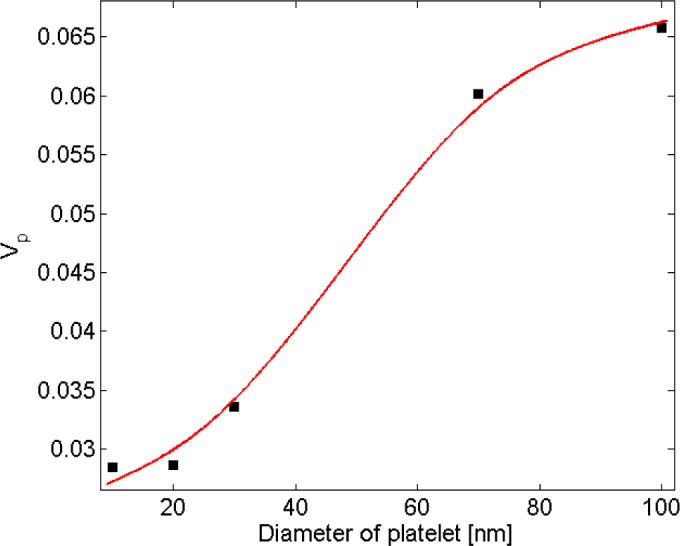
Graph depicting filler volume fraction at the percolation threshold *V*_p_ *vs*. nano-platelet diameter *D*.

Balberg *et al.* [47] showed that tunneling percolation conductivity yields a staircase effect in both lattice and continuum percolation systems. They also reported that the existence of the staircase percolation behavior is validated by experimental data [1,47–53]. Based on these findings the resistivity/filler-loading curves cannot be described by a single percolation threshold and critical exponent. In other words, Equation (1) cannot adequately describe any given set of resistivity/volume-fraction data, and each subset of data should be described with different values of *s* and *V*_p_. The present study confirmed that a single expression with specific values of *s* and *V*_p_ is insufficient to represent the entire nanocomposite conductivity and percolation behavior. This is illustrated in Figure 8, where the power-law description with a universal value of *s* (*s*_u_ *=* 2) was fitted to the resistivity data for nanocomposites with filler diameter of 20 nm. For low volume fractions the data points deviate little from the universal description, while the deviation increases with filler concentration. Interestingly, a threshold can be inferred from the curve at which the critical exponent regime changes. Findings illustrated in Figure 9 are results obtained from fitting Equation (1) to a data subset with interval of (*V*_p_, 1.4*V*_p_). Figure 10 shows the power-law approximation for higher filler volume fractions. As illustrated by Figure 11, fitting Equation (1) to the dilute region of the curve yields values of *s* with small deviation from *s*_u_. Employing the power-law equation to describe the composite behavior for higher volume fractions, on the other hand, yields a mean value of 5.56 for the critical exponent, *i.e*., *s* deviates strongly from *s*_u_. Comparing Figures 9 and 10 reveals that the conductivity/filler-loading curves describe a rather smooth behavior for volume fractions well above the percolation region, while a somewhat “erratic” behavior can be observed near the percolation threshold. Assuming a single expression for the description of nanocomposite conductivity may necessitate ignoring the percolation neighborhood and considering its data outliers. As mentioned in [47,54], close agreement between *s* and *s*_u_ is expected to be restricted to a small region near the percolation threshold, and non-universal percolation behavior becomes dominant as the filler volume fraction is increasing. Based on the above considerations, results obtained in the present study for conductive circular nano-disks indicate a critical exponent that is filler content dependent and converges toward the universal value with filler loading approaching the percolation threshold. This conclusion is congruent with findings from Johner *et al.* [55]. Data for different nano-disk sizes given in Figures 9, 10 and 12 further indicate an increasing percolation threshold and electrical conductivity with increasing nano-disks size. Note that the trendline shown in Figure 12 only illustrates the data trend.

**Figure 13.**
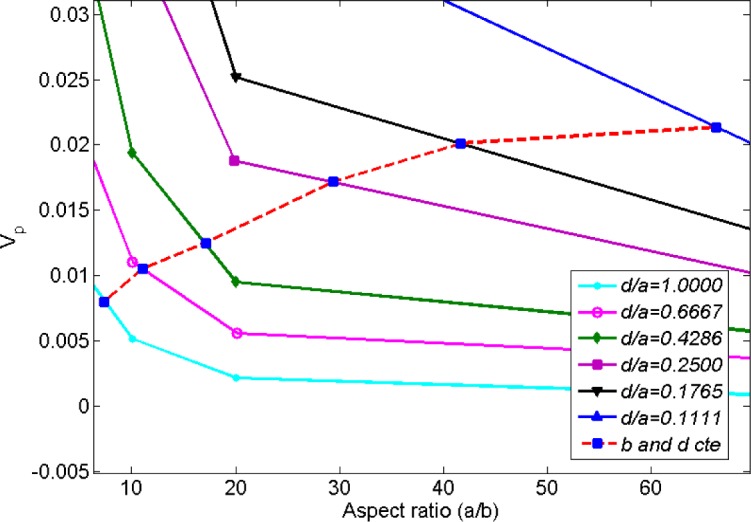
Percolation threshold of oblates of revolution as a function of aspect ratio and ratio of soft shell thickness to the major axis. The dashed line depicts the percolation threshold trend when *b* and *d* are constant. (adopted from [26]).

**Figure 14.**
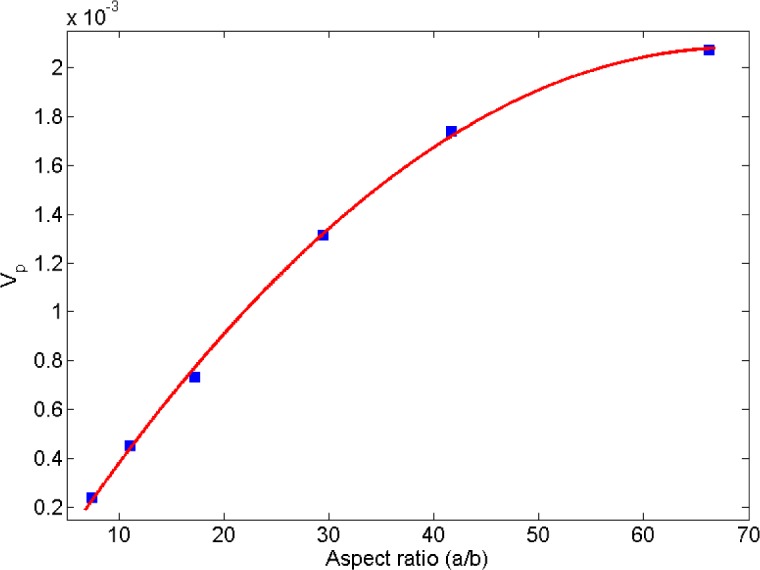
Percolation threshold of oblates of revolution as a function of aspect ratio. The soft shell volume was deducted in order to make the data analogous to a tunneling percolation problem with platelet based nanocomposites. (adopted from [26]).

Ambrosetti *et al.* [26] studied the percolation threshold for hard oblate ellipsoids of revolution surrounded by a penetrable soft shell, which is analogous to the electron tunneling concept employed in the present study. Nano-disks can be assumed as spheroids where the major axis *a* is much larger than the minor axis *b* (axis of symmetry). In [26], it was shown that the percolation threshold is governed by the ratio of the major axis to the minor axis, *a*/*b*, and the ratio of the soft shell thickness *d* to the major axis, *d*/*a*. In the present study, parameters *a*, *b* and *d* are equivalent to the nano-disk radius, thickness and tunneling distance, respectively. As illustrated in Figure 13, percolation thresholds for different oblate radii (*i.e*., 1.25, 1.88, 2.92, 5.00, 7.10, 11.25 nm) were computed keeping *b* and *d* constant at 0.17 nm (disk thickness/2) and 1.25 nm (tunneling distance/2), respectively. Disk radii were chosen to correspond to percolation thresholds provided in [26]. Critical volume fractions provided in Figure 13 include the volume of the soft penetrable shell surrounding the hard oblates. As explained previously, the soft shell is equivalent to tunneling distance concept in this study, so the volume of the soft shell has been deducted from the volume fraction provided by Figure 13 using the correction factor *a*^2^*b*/((*a* + *d*)^2^(*b* + *d*)). Resulting percolation thresholds are given in Figure 14, which indicates an increasing percolation threshold for an increasing oblate major axis (tunneling distance and oblates thickness are constant). It worth noting that in the present study the percolation threshold was evaluated for circular nano-disks, while in [26] the percolation threshold of oblates was investigated, and results from [26] are provided herein to illustrate the qualitative agreement between trends.

**Figure 15.**
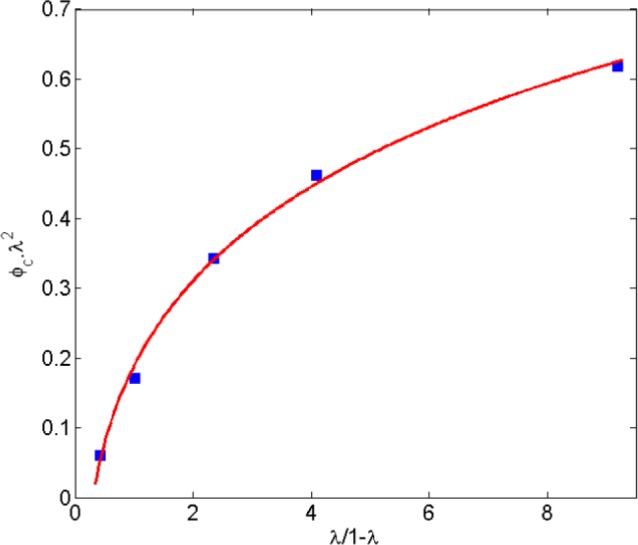
Percolation threshold for 2D disks dispersed in a 2D domain. (adopted from [56]).

In another work, Lee and Torquato [56] evaluated the critical area fraction for 2D circular disks with impenetrable radius of λ*R* surrounded by a penetrable shell with thickness (1 − λ)*R*, dispersed in a 2D domain. Similar to Ambrosetti’s work [26], (1 − λ)*R* is equivalent to the tunneling distance used in the present study. In [56] the critical area fraction is given in term of λ. In Figure 15 percolation thresholds are replotted *versus* λ/(1 − λ), a parameter representing the ratio of disk diameter to tunneling distance. Values for the percolation threshold were multiplied by λ^2^ to exclude the area of the penetrable shell that is equivalent to the tunneling distance. As illustrated by Figure 15, the percolation threshold increases as the ratio of disk size to the tunneling distance is increasing. Furthermore, Mathew *et al.* [28] showed that the dependency of platelet based conductive composite percolation threshold on the aspect ratio is in contradiction with that of needle-like fillers [57–59]. In other words, trends obtained for the relation between aspect ratio and percolation threshold of needle-like fillers cannot be generalized to a platelet-like filler percolation problem.

### 3.4. Piezoresistivity of Nano-Platelet Nano-Composites

**Figure 16.**
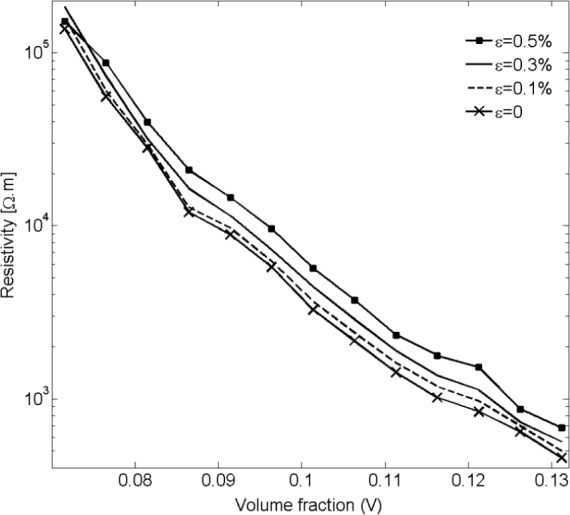
Resistivity *vs*. filler volume fraction demonstrating the response of an unconstrained nano-platelet composite to applied tensile strain *ε* for a platelet diameter of *D* = 70 nm.

Employing the methods described in Section 2.3, the piezoresistivity behavior of nanocomposites with nano-platelet inclusions was investigated. Simulation results shown in Figure 16 for unstrained nano-composites and three applied tensile strains (*ε* = 0.1%, 0.3%, 0.5%) demonstrate an increase in resistivity with applied strain.

**Figure 17.**
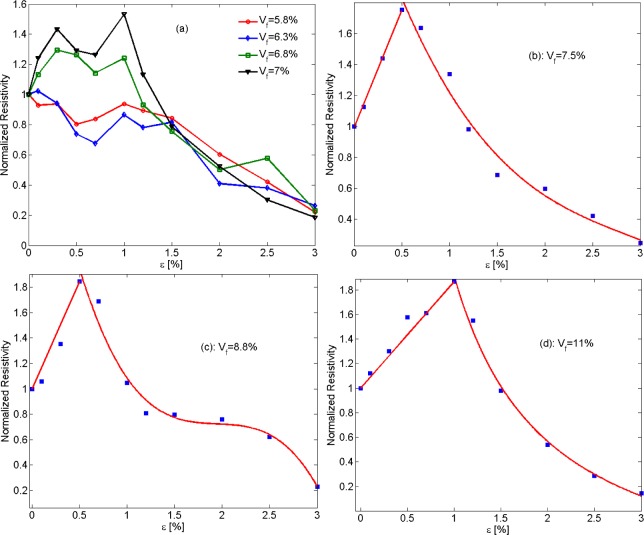
Graphs depicting the effect of filler volume fraction on piezoresistivity with tensile strain ε applied to unconstrained nano-platelet composites with particle size of *D* = 70 nm (Piezoresistivity values were normalized by dividing resistivity values by resistivity of unstrained samples). (**a**) Erratic piezoresistivity behavior for low filler loadings; (**b**) Piezoresistivity behavior for *V*_f_ = 7.5%; (**c**) Piezoresistivity graph with plateau region for *V*_f_ = 8.8%; (**d**) Piezoresistivity behavior for *V*_f_ = 11%.

In Figure 17, the piezoresistivity behavior of nano-platelet based conductive composites with four different filler volume fractions is presented. According to prediction data shown in Figure 17a, nano-composites exhibit somewhat erratic piezoresistivity behavior for low filler volume fractions, while the nanocomposite piezoresistivity behavior becomes smoother with increasing filler concentration.

**Figure 18.**
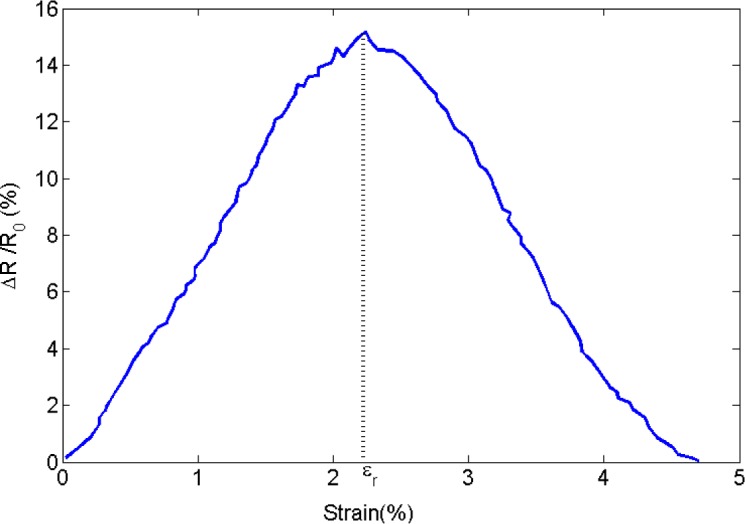
Piezoresistivity behavior and critical strain for a CNT-graphene nano-platelet hybrid composite. (adopted from [61]).

As mentioned above, piezoresistivity in conductive composites arises from two major mechanisms, that is, changing particle proximity and orientation. Increasing the tensile strain increases the average interparticular distance, so an increase in resistivity of the conductive nanocomposite is expected. On the other hand, tensile strain raises the propensity for the formation of clusters, leading to a decrease in electrical resistivity. This conjecture is supported by the results shown in Figure 17b,d. For small values of mechanical strain, piezoresistivity behavior is governed by changes in interparticular distance, causing resistivity to rise approximately linearly with strain. A critical strain can then be found at which the nanocomposite piezoresistivity behavior changes, that is, resistivity now decreases monotonically with increasing strain. Luheng *et al.* [41] reported similar behavior for silicone rubber composites filled with carbon black, *i.e*., a critical pressure was observed at which a change in the trend of the piezoresistivity effect occurred. The existence of a critical pressure was also described by Lu *et al.* [60] for the piezoresistive behavior of polyethylene nanocomposites with exfoliated graphite. Li *et al.* [61] also reported a critical strain for carbon nanotube–graphene nano-platelet hybrid composites. As illustrated by Figure 18, the electrical resistivity increased with mechanical strain up to a critical strain upon which resistivity begins to decrease with rising strain. The data presented in Figure 17b–d further reveals an increase in critical strain for increasing filler volume fraction. Additional studies are warranted to further evaluate the effect of the governing parameters such as filler size and concentration on the critical strain.

The observed “switching behavior” and the existence of a critical strain have been reported in several other studies. Kalanadhabhatla [62] conducted a comprehensive discussion and literature review on the switching piezoresistivity behavior of conducting filler based nanocomposites. Two different kinds of piezoresistivity behavior and variations of resistivity trends have been reported in the technical literature. Some studies described negative gauge factors at the onset of tensile strain application, followed by an increase in resistivity as strain is increasing. Some nanocomposites with fillers, such as carbon black [63], CNT [64,65], and graphite nanosheets [66] were found to have a positive gauge factor for lower strains, while some studies reported a decreasing resistivity up to a critical strain with increasing tensile strain [67–70]. The various studies devoted to piezoresistivity of conducting filler/polymer composites indicate that geometrical properties such as filler aspect ratio and filler concentration play a critical role in the piezoresistivity behavior of conductive nanocomposites. The role of aspect ratio on piezoresistivity behavior can be comprehended by comparing the findings in [67,69]. In [69], graphite particles with diameters between 5 and 15 μm and thicknesses ranging from 1 to 2 μm dispersed in silicone rubber yielded a decrease in resistivity for compressive strains, while in [67], silicone/graphite nanosheet specimens with filler thicknesses of 30 to 80 nm and aspect ratios of 100 to 500 showed an increase in resistivity. As illustrated by Figure 17, the sensitivity of piezoresistive nanocomposites (slope of the curve for strains lower than critical strains) is governed by filler concentration. In another study, Kost *et al.* [71] showed that carbon black based conductive composites display higher sensing sensitivity for lower filler concentrations, which is in agreement with findings in the present study. Near the percolation region there are few percolation clusters, and the formation or destruction of percolation clusters caused by strain has thus a significant contribution toward the change in nanocomposite resistivity.

It is interesting to note that for some intermediate filler volume fractions, *i.e*., volume fractions between those shown in Figure 17b, d, a diminished piezoresistivity effect can be observed for increasing strain as indicated by the plateau in the curve shown in Figure 17c. This behavior can be explained considering aforementioned piezoresistivity mechanisms in conductive nano-composites, that is, at strain levels within the plateau region none of the two-piezoresistivity mechanisms is dominant; thus their effects cancel each other, leading to diminutive piezoresistivity behavior.

## 4. Conclusions

Using a combined Monte Carlo and finite element modeling framework a theoretical study was carried out to investigate the electrical conductivity and piezoresistivity behavior of nano-composites filled with conductive nano-platelets such as graphene. The power law relationship describing the conductivity of such polymers for filler volume fractions greater than the percolation threshold was investigated and discussed. The critical exponent was approximately constant for all of the nano-disk sizes considered herein, which led to the conclusion that the exponent is controlled by filler geometry rather than size. In addition, the critical exponent in the power law description depends on the filler volume fraction. Near the percolation threshold, the exponent was found to match the often-cited value of 2, whereas the exponent deviates from this value for higher filler loadings. Modeling results further indicate a reduction in percolation threshold for decreasing nano-disks sizes and a strong effect of matrix electrical properties, *i.e*., the barrier height of the insulator. The investigation of the piezoresistivity behavior was performed through affine transformation. The filler volume fraction was found to play an important role in the piezoresitivity behavior. An erratic piezoresistivity behavior was observed for low filler volume fractions just exceeding the percolation threshold. For higher volume fractions an approximately linear correlation with strain was predicted up to a critical strain at which the resistivity behavior was found to change. For strains higher than the critical strain a monotonic decrease in electrical resistivity was observed. Overall, values of the critical strain were observed to rise with increasing filler volume fraction. Consequently, piezoresistivity behavior of nano-platelet based conductive composites can be tailored by adjusting the conductive filler content.
